# 2-[(Dodecylsulfanyl)carbonothioyl­sulfanyl]­propanoic acid

**DOI:** 10.1107/S1600536811007963

**Published:** 2011-03-09

**Authors:** Shude Xiao, Paul A. Charpentier

**Affiliations:** a Dept. of Chemical and Biochemical Engineering, Faculty of Engineering, The University of Western Ontario, London, Ontario, Canada N6A 5B9

## Abstract

In the title compound, C_16_H_30_O_2_S_3_, the decyl chain adopts an extended zigzag conformation. Two mol­ecules are disposed about a center of inversion, forming an O—H⋯O hydrogen-bonded dimer.

## Related literature

For use in polymerization of acrylic acid and acrylates, see: Alb *et al.* (2008 ▶, 2009 ▶); Konkolewicz *et al.* (2009 ▶). Various vinyl monomers can be polymerized *via* the RAFT (addition-fragmentation chain-transfer) mechanism by varying the substitutes of the trithio­carbonates, see: Moad *et al.* (2005 ▶, 2008 ▶). For related structures, see: Xiao & Charpentier (2010 ▶, 2011 ▶).
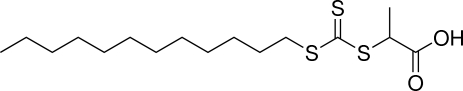


## Experimental

### 

#### Crystal data

C_16_H_30_O_2_S_3_
*M* *_r_* = 350.58
Triclinic, 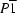

*a* = 6.5632 (4) Å
*b* = 7.0872 (4) Å
*c* = 22.0276 (14) Å
α = 85.819 (2)°
β = 86.873 (2)°
γ = 68.313 (2)°
*V* = 949.13 (10) Å^3^
*Z* = 2
Mo *K*α radiation
μ = 0.39 mm^−1^
*T* = 150 K
0.14 × 0.08 × 0.06 mm

#### Data collection

Bruker APEXII diffractometer
Absorption correction: multi-scan (*SADABS*; Sheldrick, 1996 ▶) *T*_min_ = 0.949, *T*_max_ = 0.977
40281 measured reflections
4522 independent reflections
3696 reflections with *I* > 2σ(*I*)
*R*_int_ = 0.050

#### Refinement

*R*[*F* ^2^ > 2σ(*F* ^2^)] = 0.035
*wR*(*F* ^2^) = 0.098
*S* = 1.08
4522 reflections
193 parameters
H-atom parameters constrained
Δρ_max_ = 0.48 e Å^−3^
Δρ_min_ = −0.23 e Å^−3^

### 

Data collection: *APEX2* (Bruker, 2005 ▶); cell refinement: *SAINT* (Bruker, 2005 ▶); data reduction: *SAINT*; program(s) used to solve structure: *SHELXS97* (Sheldrick, 2008 ▶); program(s) used to refine structure: *SHELXL97* (Sheldrick, 2008 ▶); molecular graphics: *SHELXTL* (Sheldrick, 2008 ▶); software used to prepare material for publication: *SHELXTL*.

## Supplementary Material

Crystal structure: contains datablocks global, I. DOI: 10.1107/S1600536811007963/ng5117sup1.cif

Structure factors: contains datablocks I. DOI: 10.1107/S1600536811007963/ng5117Isup2.hkl

Additional supplementary materials:  crystallographic information; 3D view; checkCIF report

## Figures and Tables

**Table 1:** Hydrogen-bond geometry (Å, °)

*D*—H⋯*A*	*D*—H	H⋯*A*	*D*⋯*A*	*D*—H⋯*A*
O1—H1⋯O2^i^	0.84	1.79	2.6292 (15)	175

Symmetry code: (i) 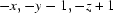.

**Fig. 1.**
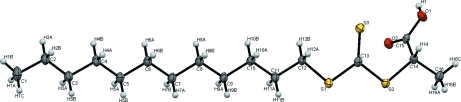
View of the title compound (50% probability displacement ellipsoids).

**Fig. 2.**
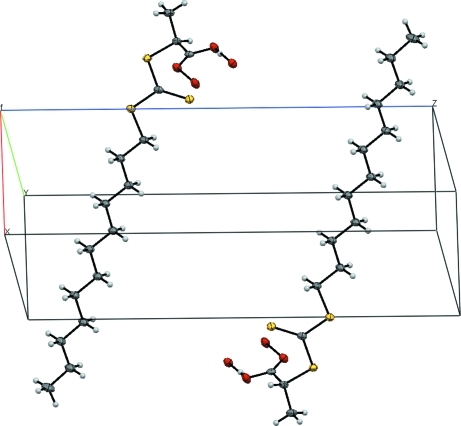
Packing diagram of the structure with H-bonds.

## supplementary crystallographic information

### Comment

Trithiocarbonates are a type of chain transfer agents (CTA) that are used in addition-fragmentation chain-transfer (RAFT) polymerization. Various vinyl monomers can be polymerized *via* the RAFT mechanism by varying the substitutes of the trithiocarbonates (Moad *et al.*, 2005, 2008). 2-(Dodecylthiocarbonothioylthio)propanoic acid was synthesized as the RAFT-CTA mainly for polymerization of acrylic acid and acrylates, but a few other vinyl monomers were also successfully polymerized, such as acrylonitrile / 1,3-butadiene and *N*-isopropylacrylamide. From solution or emulsion RAFT polymerization, diblock and triblock copolymers were prepared from acrylates/acrylic acid and other vinyl monomers. Via its carboxylic acid group, 2-(dodecylthiocarbonothioylthio)propanoic acid was immobilized onto nanoparticles, such as SiO_2_ and carbon black, followed by RAFT polymerization yielding hybrid nanocomposites.

### Experimental

1-Dodecanethiol 20 g (0.1 mol), triethylamine 12 g (0.12 mol) were mixed in THF 30 ml, and then carbon disulfide 11 g was added into the mixture dropwise at room temperature. The mixture was kept stirred for 1 day, and 2- bromopropionic acid 15.3 g (0.1 mol) / THF 5 ml were charged into it. The reaction lasted for 2 days at room temperature. Excess ethyl ether was used to precipitate the salts, and the solvents were evaporated. The crude product was treated with hydrobromic acid followed by extraction with ethyl ether. When the solvents were being removed, toluene was added to get rid of the residual water. Yellow crystals of 2-(dodecylthiocarbonothioylthio)propanoic acid were obtained from recrystalization in hexane/cyclohexane (10:1). m.p.: 77.21°C (DSC).

### Refinement

Hydrogen atom positions were calculated geometrically and were included as riding on their respective carbon/oxygen atoms.

### Crystal data

**Table:**

C_16_H_30_O_2_S_3_	*Z* = 2
*M**_r_* = 350.58	*F*(000) = 380
Triclinic, *P*1	*D*_x_ = 1.227 Mg m^−^^3^
Hall symbol: -P 1	Mo *K*α radiation, λ = 0.71073 Å
*a* = 6.5632 (4) Å	Cell parameters from 8106 reflections
*b* = 7.0872 (4) Å	θ = 3.1–28.2°
*c* = 22.0276 (14) Å	µ = 0.39 mm^−^^1^
α = 85.819 (2)°	*T* = 150 K
β = 86.873 (2)°	Block, yellow
γ = 68.313 (2)°	0.14 × 0.08 × 0.06 mm
*V* = 949.13 (10) Å^3^	

### Data collection

**Table:**

Bruker APEXII diffractometer	4522 independent reflections
Radiation source: fine-focus sealed tube	3696 reflections with *I* > 2σ(*I*)
graphite	*R*_int_ = 0.050
φ and ω scans	θ_max_ = 27.9°, θ_min_ = 1.9°
Absorption correction: multi-scan (*SADABS*; Sheldrick, 1996)	*h* = −7→8
*T*_min_ = 0.949, *T*_max_ = 0.977	*k* = −9→9
40281 measured reflections	*l* = −28→28

### Refinement

**Table:**

Refinement on *F*^2^	Primary atom site location: structure-invariant direct methods
Least-squares matrix: full	Secondary atom site location: difference Fourier map
*R*[*F*^2^ > 2σ(*F*^2^)] = 0.035	Hydrogen site location: inferred from neighbouring sites
*wR*(*F*^2^) = 0.098	H-atom parameters constrained
*S* = 1.08	*w* = 1/[σ^2^(*F*_o_^2^) + (0.0524*P*)^2^ + 0.166*P*] where *P* = (*F*_o_^2^ + 2*F*_c_^2^)/3
4522 reflections	(Δ/σ)_max_ = 0.002
193 parameters	Δρ_max_ = 0.48 e Å^−^^3^
0 restraints	Δρ_min_ = −0.23 e Å^−^^3^

### Special details

**Table:**

Geometry. All e.s.d.'s (except the e.s.d. in the dihedral angle between two l.s. planes) are estimated using the full covariance matrix. The cell e.s.d.'s are taken into account individually in the estimation of e.s.d.'s in distances, angles and torsion angles; correlations between e.s.d.'s in cell parameters are only used when they are defined by crystal symmetry. An approximate (isotropic) treatment of cell e.s.d.'s is used for estimating e.s.d.'s involving l.s. planes.
Refinement. Refinement of *F*^2^ against ALL reflections. The weighted *R*-factor *wR* and goodness of fit *S* are based on *F*^2^, conventional *R*-factors *R* are based on *F*, with *F* set to zero for negative *F*^2^. The threshold expression of *F*^2^ > σ(*F*^2^) is used only for calculating *R*-factors(gt) *etc*. and is not relevant to the choice of reflections for refinement. *R*-factors based on *F*^2^ are statistically about twice as large as those based on *F*, and *R*- factors based on ALL data will be even larger.

### Fractional atomic coordinates and isotropic or equivalent isotropic displacement parameters (Å^2^)

**Table:**

	*x*	*y*	*z*	*U*_iso_*/*U*_eq_	
S1	−0.09326 (7)	0.13333 (7)	0.295324 (18)	0.02645 (12)	
S2	−0.35456 (6)	−0.07612 (6)	0.347313 (17)	0.02119 (11)	
S3	−0.15639 (7)	0.12153 (6)	0.432049 (18)	0.02365 (11)	
O1	−0.27164 (17)	−0.31220 (17)	0.51448 (5)	0.0248 (3)	
H1	−0.1628	−0.3995	0.5317	0.037*	
O2	−0.05484 (17)	−0.39868 (16)	0.43093 (5)	0.0217 (2)	
C1	1.3203 (3)	1.3296 (3)	0.02501 (9)	0.0390 (5)	
H1A	1.4290	1.2072	0.0081	0.059*	
H1B	1.3941	1.4182	0.0369	0.059*	
H1C	1.2121	1.4022	−0.0058	0.059*	
C2	1.2050 (3)	1.2690 (3)	0.08064 (8)	0.0287 (4)	
H2A	1.3149	1.2010	0.1121	0.034*	
H2B	1.0971	1.3938	0.0976	0.034*	
C3	1.0870 (3)	1.1280 (3)	0.06765 (7)	0.0250 (4)	
H3A	1.1945	1.0026	0.0509	0.030*	
H3B	0.9766	1.1956	0.0363	0.030*	
C4	0.9730 (3)	1.0703 (2)	0.12409 (7)	0.0239 (3)	
H4A	0.8645	1.1960	0.1404	0.029*	
H4B	1.0835	1.0055	0.1556	0.029*	
C5	0.8561 (3)	0.9263 (2)	0.11290 (7)	0.0236 (3)	
H5A	0.9641	0.7998	0.0969	0.028*	
H5B	0.7451	0.9906	0.0815	0.028*	
C6	0.7434 (3)	0.8722 (2)	0.17038 (7)	0.0227 (3)	
H6A	0.8554	0.8058	0.2014	0.027*	
H6B	0.6385	0.9994	0.1869	0.027*	
C7	0.6207 (3)	0.7320 (2)	0.16040 (7)	0.0230 (3)	
H7A	0.5085	0.7980	0.1294	0.028*	
H7B	0.7254	0.6042	0.1442	0.028*	
C8	0.5090 (3)	0.6809 (2)	0.21833 (7)	0.0226 (3)	
H8A	0.6212	0.6164	0.2494	0.027*	
H8B	0.4036	0.8088	0.2343	0.027*	
C9	0.3876 (3)	0.5393 (2)	0.20916 (7)	0.0221 (3)	
H9A	0.4929	0.4109	0.1935	0.027*	
H9B	0.2755	0.6034	0.1780	0.027*	
C10	0.2757 (3)	0.4903 (2)	0.26735 (7)	0.0218 (3)	
H10A	0.1689	0.6182	0.2829	0.026*	
H10B	0.3871	0.4267	0.2987	0.026*	
C11	0.1569 (3)	0.3471 (2)	0.25700 (7)	0.0218 (3)	
H11A	0.2634	0.2199	0.2410	0.026*	
H11B	0.0444	0.4114	0.2260	0.026*	
C12	0.0468 (3)	0.2959 (2)	0.31527 (7)	0.0210 (3)	
H12A	−0.0591	0.4217	0.3323	0.025*	
H12B	0.1579	0.2249	0.3461	0.025*	
C13	−0.1976 (2)	0.0663 (2)	0.36428 (7)	0.0183 (3)	
C14	−0.4285 (2)	−0.1630 (2)	0.42127 (7)	0.0183 (3)	
H14	−0.5124	−0.0434	0.4456	0.022*	
C15	−0.2289 (2)	−0.3009 (2)	0.45571 (7)	0.0174 (3)	
C16	−0.5746 (3)	−0.2836 (3)	0.41072 (8)	0.0262 (4)	
H16A	−0.4925	−0.3999	0.3865	0.039*	
H16B	−0.7051	−0.1953	0.3889	0.039*	
H16C	−0.6195	−0.3323	0.4501	0.039*	

### Atomic displacement parameters (Å^2^)

**Table:**

	*U*^11^	*U*^22^	*U*^33^	*U*^12^	*U*^13^	*U*^23^
S1	0.0348 (2)	0.0340 (2)	0.0210 (2)	−0.0251 (2)	0.00321 (17)	−0.00228 (16)
S2	0.0230 (2)	0.0240 (2)	0.02111 (19)	−0.01424 (17)	−0.00368 (15)	0.00279 (15)
S3	0.0282 (2)	0.0258 (2)	0.0219 (2)	−0.01563 (17)	0.00128 (16)	−0.00304 (15)
O1	0.0193 (6)	0.0284 (6)	0.0204 (5)	−0.0024 (5)	0.0014 (4)	0.0034 (5)
O2	0.0189 (6)	0.0198 (5)	0.0231 (5)	−0.0038 (5)	0.0022 (4)	−0.0004 (4)
C1	0.0373 (11)	0.0417 (11)	0.0447 (11)	−0.0248 (9)	0.0076 (9)	0.0050 (9)
C2	0.0302 (9)	0.0303 (9)	0.0310 (9)	−0.0182 (8)	0.0051 (7)	−0.0013 (7)
C3	0.0268 (9)	0.0269 (8)	0.0253 (8)	−0.0152 (7)	0.0034 (7)	0.0000 (7)
C4	0.0260 (8)	0.0225 (8)	0.0266 (8)	−0.0134 (7)	0.0046 (7)	−0.0021 (6)
C5	0.0249 (8)	0.0253 (8)	0.0246 (8)	−0.0147 (7)	0.0012 (6)	0.0013 (6)
C6	0.0230 (8)	0.0231 (8)	0.0253 (8)	−0.0129 (7)	0.0016 (6)	0.0003 (6)
C7	0.0226 (8)	0.0242 (8)	0.0261 (8)	−0.0136 (7)	0.0015 (6)	−0.0010 (6)
C8	0.0220 (8)	0.0241 (8)	0.0253 (8)	−0.0132 (7)	0.0006 (6)	0.0005 (6)
C9	0.0220 (8)	0.0227 (8)	0.0254 (8)	−0.0131 (7)	0.0020 (6)	−0.0006 (6)
C10	0.0216 (8)	0.0229 (8)	0.0240 (8)	−0.0122 (7)	0.0007 (6)	−0.0010 (6)
C11	0.0236 (8)	0.0215 (8)	0.0236 (8)	−0.0125 (7)	0.0035 (6)	−0.0017 (6)
C12	0.0217 (8)	0.0238 (8)	0.0222 (7)	−0.0142 (7)	0.0028 (6)	−0.0020 (6)
C13	0.0144 (7)	0.0151 (7)	0.0241 (7)	−0.0042 (6)	0.0001 (6)	0.0010 (6)
C14	0.0153 (7)	0.0178 (7)	0.0218 (7)	−0.0065 (6)	−0.0004 (6)	0.0006 (6)
C15	0.0167 (7)	0.0150 (7)	0.0233 (7)	−0.0092 (6)	−0.0003 (6)	−0.0005 (6)
C16	0.0216 (8)	0.0287 (9)	0.0327 (9)	−0.0147 (7)	−0.0010 (7)	0.0007 (7)

### Geometric parameters (Å, °)

**Table:**

S1—C13	1.7388 (15)	C6—H6A	0.9900
S1—C12	1.8073 (15)	C6—H6B	0.9900
S2—C13	1.7565 (15)	C7—C8	1.525 (2)
S2—C14	1.8047 (15)	C7—H7A	0.9900
S3—C13	1.6316 (16)	C7—H7B	0.9900
O1—C15	1.3126 (18)	C8—C9	1.523 (2)
O1—H1	0.8400	C8—H8A	0.9900
O2—C15	1.2189 (18)	C8—H8B	0.9900
C1—C2	1.523 (2)	C9—C10	1.524 (2)
C1—H1A	0.9800	C9—H9A	0.9900
C1—H1B	0.9800	C9—H9B	0.9900
C1—H1C	0.9800	C10—C11	1.525 (2)
C2—C3	1.523 (2)	C10—H10A	0.9900
C2—H2A	0.9900	C10—H10B	0.9900
C2—H2B	0.9900	C11—C12	1.525 (2)
C3—C4	1.524 (2)	C11—H11A	0.9900
C3—H3A	0.9900	C11—H11B	0.9900
C3—H3B	0.9900	C12—H12A	0.9900
C4—C5	1.525 (2)	C12—H12B	0.9900
C4—H4A	0.9900	C14—C15	1.516 (2)
C4—H4B	0.9900	C14—C16	1.537 (2)
C5—C6	1.528 (2)	C14—H14	1.0000
C5—H5A	0.9900	C16—H16A	0.9800
C5—H5B	0.9900	C16—H16B	0.9800
C6—C7	1.525 (2)	C16—H16C	0.9800
			
C13—S1—C12	104.56 (7)	C7—C8—H8A	108.8
C13—S2—C14	103.56 (7)	C9—C8—H8B	108.8
C15—O1—H1	109.5	C7—C8—H8B	108.8
C2—C1—H1A	109.5	H8A—C8—H8B	107.7
C2—C1—H1B	109.5	C8—C9—C10	113.18 (13)
H1A—C1—H1B	109.5	C8—C9—H9A	108.9
C2—C1—H1C	109.5	C10—C9—H9A	108.9
H1A—C1—H1C	109.5	C8—C9—H9B	108.9
H1B—C1—H1C	109.5	C10—C9—H9B	108.9
C3—C2—C1	114.08 (15)	H9A—C9—H9B	107.8
C3—C2—H2A	108.7	C9—C10—C11	112.08 (13)
C1—C2—H2A	108.7	C9—C10—H10A	109.2
C3—C2—H2B	108.7	C11—C10—H10A	109.2
C1—C2—H2B	108.7	C9—C10—H10B	109.2
H2A—C2—H2B	107.6	C11—C10—H10B	109.2
C2—C3—C4	112.89 (14)	H10A—C10—H10B	107.9
C2—C3—H3A	109.0	C10—C11—C12	112.22 (13)
C4—C3—H3A	109.0	C10—C11—H11A	109.2
C2—C3—H3B	109.0	C12—C11—H11A	109.2
C4—C3—H3B	109.0	C10—C11—H11B	109.2
H3A—C3—H3B	107.8	C12—C11—H11B	109.2
C3—C4—C5	114.28 (13)	H11A—C11—H11B	107.9
C3—C4—H4A	108.7	C11—C12—S1	107.01 (10)
C5—C4—H4A	108.7	C11—C12—H12A	110.3
C3—C4—H4B	108.7	S1—C12—H12A	110.3
C5—C4—H4B	108.7	C11—C12—H12B	110.3
H4A—C4—H4B	107.6	S1—C12—H12B	110.3
C4—C5—C6	112.82 (13)	H12A—C12—H12B	108.6
C4—C5—H5A	109.0	S3—C13—S1	127.02 (9)
C6—C5—H5A	109.0	S3—C13—S2	126.17 (9)
C4—C5—H5B	109.0	S1—C13—S2	106.81 (8)
C6—C5—H5B	109.0	C15—C14—C16	108.83 (12)
H5A—C5—H5B	107.8	C15—C14—S2	112.00 (10)
C7—C6—C5	114.09 (13)	C16—C14—S2	107.08 (11)
C7—C6—H6A	108.7	C15—C14—H14	109.6
C5—C6—H6A	108.7	C16—C14—H14	109.6
C7—C6—H6B	108.7	S2—C14—H14	109.6
C5—C6—H6B	108.7	O2—C15—O1	124.51 (14)
H6A—C6—H6B	107.6	O2—C15—C14	123.51 (14)
C8—C7—C6	113.13 (13)	O1—C15—C14	111.83 (12)
C8—C7—H7A	109.0	C14—C16—H16A	109.5
C6—C7—H7A	109.0	C14—C16—H16B	109.5
C8—C7—H7B	109.0	H16A—C16—H16B	109.5
C6—C7—H7B	109.0	C14—C16—H16C	109.5
H7A—C7—H7B	107.8	H16A—C16—H16C	109.5
C9—C8—C7	113.71 (13)	H16B—C16—H16C	109.5
C9—C8—H8A	108.8		

### Hydrogen-bond geometry (Å, °)

**Table:**

*D*—H···*A*	*D*—H	H···*A*	*D*···*A*	*D*—H···*A*
O1—H1···O2^i^	0.84	1.79	2.6292 (15)	175

Symmetry codes: (i) −*x*, −*y*−1, −*z*+1.
